# Isoniazid-specific T-cell Responses in patients with anti-tuberculosis drug related liver injury

**DOI:** 10.1186/2045-7022-4-S3-P116

**Published:** 2014-07-18

**Authors:** Toru Usui, Katy Saide, Xiaoli Meng, Paul Whitaker, Daniel Peckham, Neil French, Dean Naisbitt, Kevin Park

**Affiliations:** 1 University of Liverpool, MRC Centre for Drug Safety Science, UK; 2 St James's Hospital, Regional Adult Cystic Fibrosis Unit, UK

## Background

A combination of isoniazid (INH), rifampicin, pyrazinamide and/or ethambutol (anti-tuberculosis drugs, ATDs) is commonly used for the treatment of tuberculosis. A significant clinical problem is that drug treatment is associated with mild elevations of liver enzymes that occasionally develop into severe liver injury. The culprit drug(s) and mechanistic basis of the reaction has not been defined. Thus, the aims of this study were to (1) explore whether drug-responsive T-lymphocytes were detectable in patients with ATD-related liver injury, (2) identify the drug(s) that activate T-cells and (3) characterize the nature of the T-cell response.

## Methods

A lymphocyte transformation test (LTT) and IFN-ELISpot assay using ATDs and isonictinic acid (INA)-human serum albumin (HSA) were performed on PBMCs from 20 ATD-related liver injury patients. HSA was coupled with INA using synthesized N-hydroxysuccinimide activated ester of INA. Subsequently, patients were selected to attempt to generate INH, rifampicin, pyrazinamide and ethambutol-specific T-cell clones by serial dilution. Any drug-responsive clones were then characterized in terms of function and mechanism of antigen presentation. Antigen present cells (APC) were (1) omitted from the assay, (2) fixed with glutaraldehyde, (3) pulsed with INH, (4) treated with MHC blocking antibodies and (5) cultured with INH in the presence of N--acetyl-l-lysine (NAL) or other reactive metabolites-trapping agents.

## Results

PBMCs from 5 patients proliferated weakly after in vitro stimulation with INH and INH-specific PBMC responses were detectable using an IFN-ELISpot. INH, but not rifampicin, pyrazinamide or ethambutol, specific T-cell clones were generated from blood of the patients. The clones proliferated and secreted cytokines when stimulated with INH alone. They responded to APC pulsed with INH for 16h and the response was inhibited by APC fixation and with an anti-HLA class II blocking antibody. However, NAL or other reactive metabolites-trapping agents did not inhibit INH-specific proliferation.

## Conclusion

These studies identify INH-specific T-cells in the blood of certain patients with ATD-related liver injury and characterize the nature of the drug-specific T-cell response. Further experiments are needed to define the way in which the drug interacts with MHC molecules to stimulate T-cells.

